# Machine learning-derived gut microbiome signature predicts fatty liver disease in the presence of insulin resistance

**DOI:** 10.1038/s41598-022-26102-4

**Published:** 2022-12-17

**Authors:** Baeki E. Kang, Aron Park, Hyekyung Yang, Yunju Jo, Tae Gyu Oh, Seung Min Jeong, Yosep Ji, Hyung‐Lae Kim, Han‐Na Kim, Johan Auwerx, Seungyoon Nam, Cheol-Young Park, Dongryeol Ryu

**Affiliations:** 1 Department of Molecular Cell Biology, Sungkyunkwan University School of Medicine, 2066, Seobu-Ro, Suwon, 16419 Republic of Korea; 2 Department of Health Sciences and Technology, Gachon Advanced Institute for Health Sciences and Technology, Gachon University, Incheon, 21999 Republic of Korea; 3 Medical Research Institute, School of Medicine, Kangbuk Samsung Hospital, Sungkyunkwan University, Seoul, 03181 Republic of Korea; 4 Gene Expression Laboratory, Salk Institute for Biological Studies, La Jolla, CA 92037 USA; 5 HEM Inc., 404, Ace Gwanggyo Tower 3, Suwon, 16229 Republic of Korea; 6 Department of Biochemistry, College of Medicine, Ewha Womans University, Seoul, 07985 Republic of Korea; 7 Department of Clinical Research Design and Evaluation, Samsung Advanced Institute for Health Sciences and Technology, Sungkyunkwan University, Seoul, 06355 Republic of Korea; 8 Institute of Bioengineering, Faculty of Life Sciences, Ecole Polytechnique Fédérale de Lausanne, 1015 Lausanne, Switzerland; 9 Department of Genome Medicine and Science, AI Convergence, Center for Medical Science, Gachon Institute of Genome Medicine and Science, Gachon University Gil Medical Centre, Gachon University College of Medicine, 38-13, Dokjeom-Ro 3Beon-Gil, Incheon, 21999 Republic of Korea; 10 Division of Endocrinology and Metabolism, Department of Internal Medicine, Kangbuk Samsung Hospital, Sungkyunkwan University School of Medicine, 29, Saemunan-Ro, Jongno-Gu, Seoul, 03181 Republic of Korea; 11 Biomedical Institute for Convergence at SKKU (BICS), Sungkyunkwan University, Suwon, 16419 Republic of Korea

**Keywords:** Biomarkers, Hepatology, Endocrine system and metabolic diseases, Microbiology, Gastroenterology

## Abstract

A simple predictive biomarker for fatty liver disease is required for individuals with insulin resistance. Here, we developed a supervised machine learning-based classifier for fatty liver disease using fecal 16S rDNA sequencing data. Based on the Kangbuk Samsung Hospital cohort (n = 777), we generated a random forest classifier to predict fatty liver diseases in individuals with or without insulin resistance (n = 166 and n = 611, respectively). The model performance was evaluated based on metrics, including accuracy, area under receiver operating curve (AUROC), kappa, and F1-score. The developed classifier for fatty liver diseases performed better in individuals with insulin resistance (AUROC = 0.77). We further optimized the classifiers using genetic algorithm. The improved classifier for insulin resistance, consisting of ten microbial genera, presented an advanced classification (AUROC = 0.93), whereas the improved classifier for insulin-sensitive individuals failed to distinguish participants with fatty liver diseases from the healthy. The classifier for individuals with insulin resistance was comparable or superior to previous methods predicting fatty liver diseases (accuracy = 0.83, kappa = 0.50, F1-score = 0.89), such as the fatty liver index. We identified the ten genera as a core set from the human gut microbiome, which could be a diagnostic biomarker of fatty liver diseases for insulin resistant individuals. Collectively, these findings indicate that the machine learning classifier for fatty liver diseases in the presence of insulin resistance is comparable or superior to commonly used methods.

## Introduction

Fatty liver disease (FL), or hepatic steatosis, is diagnosed when a liver has at least 5% hepatocytes containing fat^1^. Depending on its etiology, it can be categorized into alcoholic fatty liver disease (AFLD) or nonalcoholic FL disease (NAFLD). The international prevalence of NAFLD was estimated to be 25.2%^2^. However, it was estimated to be 55.5% in participants with type 2 diabetes mellitus (T2DM)^3^. Insulin resistance (IR), a major pathological contributor to T2DM and metabolic syndrome, is a potential driver of NAFLD progression in nonalcoholic steatohepatitis (NASH)^4,5^. IR is a common pathological factor of NASH and T2DM. It may also be involved in hepatocellular carcinoma (HCC) development^6^.

Liver biopsy or magnetic resonance imaging/spectroscopy (MRI/MRS) is the gold standard for diagnosing FL^7^. However, it has certain disadvantages. For instance, liver biopsy is invasive, and the examination is spatially limited to the sample. MRI/MRS is inaccessible and expensive. The most popular method for FL diagnosis is ultrasonography (USG). However, it also has several limitations: (i) variability depending on the spots observed, (ii) low sensitivity in mild hepatic steatosis occupying < 30% of the liver^8^; and (iii) inconsistencies in diagnosis between interpreters. Therefore, an additional non-invasive diagnostic biomarker for FL is required urgently.

The symbiosis or dysbiosis between the human gut microbiome (HGM) and hosts is linked to the health or disease of the host, respectively^9^. According to recent reports, alterations in the gut microbiome could be a driver of obesity and IR^10–12^. It has also been shown that specific signatures of the HGM can serve as biomarkers of liver diseases, including NASH, liver fibrosis, and cirrhosis^13–15^. However, only a few studies have proposed the HGM as a biomarker for general FL rather than for advanced liver diseases. Most studies investigated either the correlation or causality of HGM with liver diseases only in a pathological cohort rather than a healthy one. Furthermore, those studies did not invest in participants with IR, who have a higher risk for advanced FL disease, in the healthy cohort. Therefore, our study established a potential gut microbiome to identify the FL of participants with IR in a healthy cohort using supervised machine learning (ML) methods for classification, such as random forest (RF), gradient boosting machine (GBM), extreme gradient boosting (XGB) algorithm, along with genetic algorithm (GA), a random-based algorithm inspired by natural selection in biology to obtain the optimized solution^16^.

## Materials and methods

### Human participants and data collection

Stool samples were collected from the study participants (n = 1,463) from the routine annual comprehensive physical examination of the Kangbuk Samsung Health (KSH) cohort. The study participants underwent extensive periodic PE between June and September 2014^17^ and 213 participants were excluded from 1,463 participants because of missing data and poor detection. FL was diagnosed using abdominal USG with a 3.5-MHz transducer based on conventionally captured images by trained radiologists who were blinded to the study's predetermined parameters as previously described^18,19^. In the diagnosis, the inter-observer reliability value was Cohen’s kappa coefficient of 0.74, and the intra-observer reliability value was 0.94^20^.

The Institutional Review Board of Kangbuk Samsung Hospital authorized the study's protocol (2019-05-015). All participants signed a written informed consent form after being informed of possible outcomes and the nature of the study. In the study, we obeyed all applicable regulations of institutions and governments regarding human research ethics for participants, following the guidelines of the Declaration of Helsinki^21^.

### DNA purification and 16S rDNA gene sequencing

The Illumina MiSeq platform was used to sequence the fecal DNA samples, following the provided protocol (Illumina, San Diego, CA, USA)^22^. The DADA2 plugin of the QIIME 2 package (v.2020.8) was utilized in filtering out chimeras and low-quality sequences and to produce amplicon sequence variants (ASVs)^23,24^. The naïve Bayes classifier were trained, and the classifier was used to assign ASVs to microbial taxonomy against the SILVA 132 with a 99% operational taxonomy unit dataset. All 16S rDNA gene sequencing files are available in the Clinical & Omics Data Archive of the Korea National Institute of Health (accession number: R000635).

### Development of ML classifier and evaluation

R Package “caret” v.6.0-86. was used for ML approach using three ML algorithms (RF, GBM, and XGB)^25^. The RF parameter options were set to default option in the ML approach. In the GBM models’ hyperparameter settings, 10, 20, 30, 40, and 50 were used as “n.trees”; one, two, three, and four as “interaction.depth”; 0.01 and 0.001 as “shrinkage”; three, five, seven, and nine as “n.minobsinnode.” For the hyperparameter setting in the XGB models, 10, 20, 30, 40, and 50 was used as “nrounds”; three, five, seven, and nine as “max_depth”; 0.01 and 0.2 as “eta”; 0.01 as “gamma”; 0.75 as “colsample_by_tree”; 1 as “min_child_weight”; and 0.5 as “subsample.” The microbiome dataset was randomly partitioned into training (80%) and test (20%) datasets using the createDataPartition function. The dataset was preprocessed using the zv, scale, and center methods of the training function. The Synthetic Minority Over-sampling Technique (SMOTE) function in the R package “smotefamily” (v.1.3.1) was used to handle the sample imbalance issue. The tenfold three-times repeated cross-validation were applied to the training dataset for ML-based development into the previously described classification model^13^. The sequential feature selection was conducted based on the Gini importance of the features. The performance of the developed classifiers was assessed using the area under the receiver operating curve (AUROC), representing their sensitivity and specificity in the training dataset. Using the test dataset, the classifiers were evaluated using AUROC, accuracy, F1-score, and kappa.

### The optimal feature selection by GA

For a GA-based optimal feature selection, 300 individuals were randomly generated as the initial population to be sequentially evolved further by GA. The individuals carry a specific number of genera (described as “genes”) randomly selected from 87 gut microbial genera detected in the fecal samples. The selected genera were encoded as one, and the other genera were encoded as 0 in individuals to be evolved by GA. Each individual in the population was evaluated using a fitness score as follows:

1 $$ Fitness\, score = 100 \times \frac{{\mathop \sum \nolimits_{k = 1}^{M} S_{k} }}{M} - {\text{W}} \times \left| {x - b} \right| $$

where *S*_*k*_ is the AUROC score from the RF model in the *k*-th fold during *M* fold cross-validation; *x* is the number of genera selected by the RF; *W* is a penalty weight; and *b* ∈ {6, 7, 8, 9, 10} is the optimal number of biomarker genera. *M* was set to 3 and *W* to 10.

According to the fitness score, GA repeatedly searches for the best solution for classifying every generation. Firstly, in the initial population, GA selected the fittest individual with the highest fitness score in the initial population (first generation). To generate the population of the next generation, the fittest individual of the previous generation is kept, while the other individuals of the previous generation are influenced by crossovers and mutations, resulting in different individuals. Thus, we obtained 300 individuals in each generation. Then, these steps were iterated 100 times (generations) to get the best solution through the entire generation.

Package “DEAP” v.1.3.1 under python 3.7.1 was used for the GA simulations, revealing the optimal genera having the best fitness. The optimal genera served as the features of an optimal RF classification model. The crossover rate, mutation rate, and generations for GA simulations were set at 0.8, 0.003, and 300, respectively.

### Data visualization and statistical analyses

All statistical analysis and visualizations were conducted using RStudio with R v.4.1. The normality of the overall data was analyzed using Shapiro’s test. Statistical significance was computed with either two-tailed Wilcoxon’s test or Kruskal–Wallis test upon the normality and distribution, and a *p*-value < 0.05 was deemed statistically significant. The R package “ggplot2” and “pROC” were used for data visualization.

### Evaluation of model performance

To evaluate the model performance, we obtained a 2 × 2 confusion matrix from each classifier using the test dataset and calculated true positive, true negative, false positive, and false negative using predicted and observed classes. Then, we calculated and adopted four metrics for the model’s performance evaluation: accuracy^26,27^, F1-score^27^, kappa index^28^, and AUROC, plotted using true positive rate and false positive rate (FPR)^29^.

## Results

### Data processing in the healthy and FL cohort

**Figure 1:**
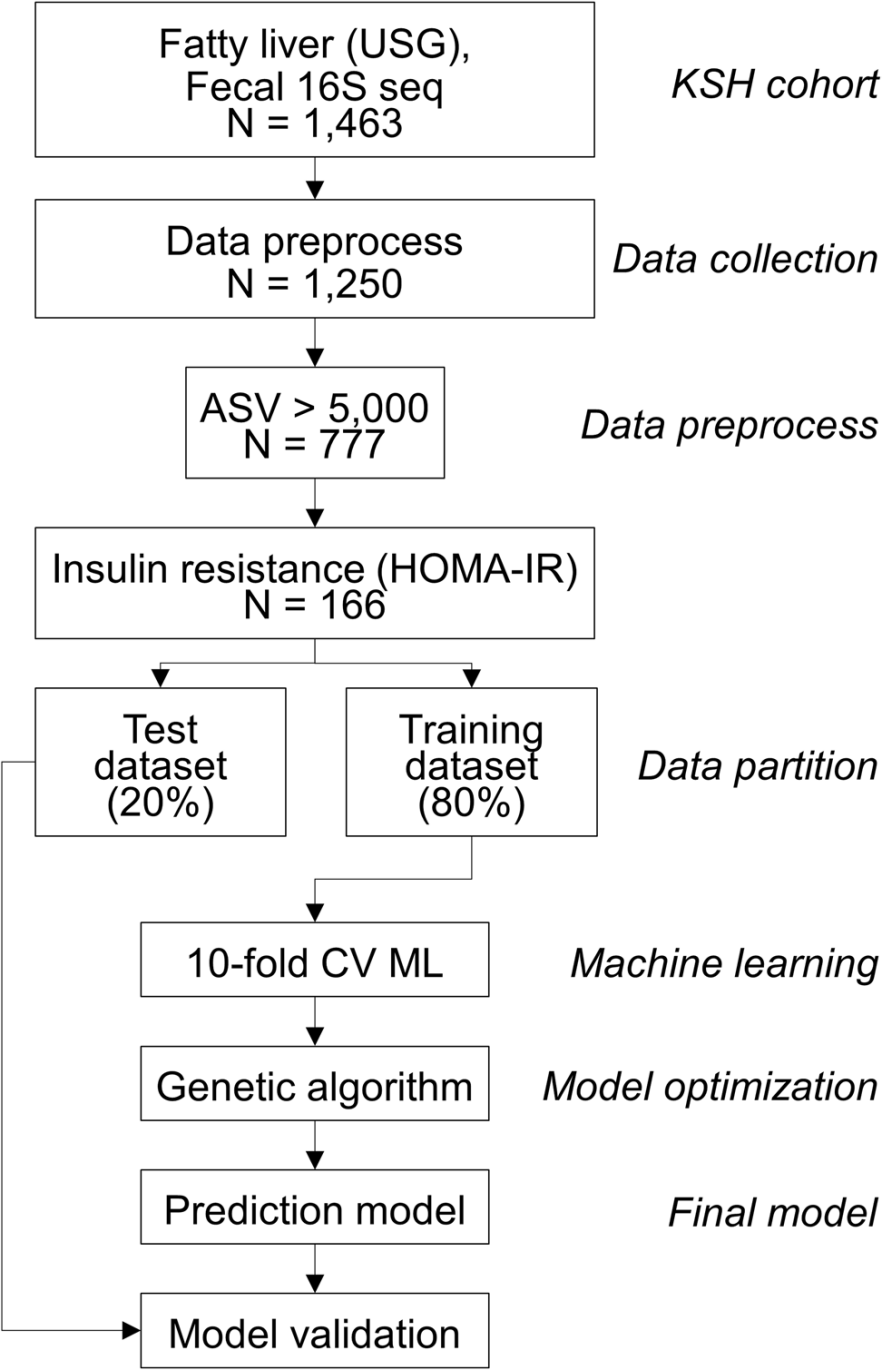
Schematic of the analysis pipeline. Participants (n = 777) from the KSH cohort were included in the final analysis and were divided into four subgroups, ISNF (n = 449), ISFL (n = 162), IRNF (n = 38), and IRFL (n = 128). ASV: amplicon sequence variants; HOMA-IR: homeostatic model assessment of insulin resistance index; IR: insulin resistant; KSH cohort: Kangbuk Samsung Hospital cohort.

Physical examination data, including high throughput 16S rDNA sequencing results from 1463 participants of KSH cohorts, were collected for the study. After removing missing and poorly detected values, 1,250 participants were included in the analysis. Subsequently, participants whose ASV number were < 5 000 were filtered out, leaving only 777 participants for the study (Fig. 1). It was revealed that 290 of the 777 participants had FL, while the remaining 487 did not. We regarded participants as insulin resistant or sensitive if their values for homeostatic model assessment of IR (HOMA-IR) were over the following cutoff: 1.8 for men and 2.2 for women, calculated as the critical threshold for T2DM development based on the KSH cohort internal investigation (data not shown). Among the 777 participants, 611 were classified into the insulin-sensitive (IS) group, while 166 were classified into the IR group based on the criteria for insulin resistance. The biological and physical characteristics of these groups are described in Table 1.

**Table 1:** Characteristics of participants in the Kangbuk Samsung Health cohort.

Characteristics	Group		*P*-value
	Insulin sensitivity	Insulin resistance	
Number	611	166	
Male (%)	55.97	84.94	
Age (years)	44.86 ± 8.783	46.94 ± 8.526	0.007
BMI (kg/m^2^)	22.87 ± 2.688	26.25 ± 3.062	< 0.001
Waist circumference (cm)	80.27 ± 8.219	89.98 ± 7.709	< 0.001
Heart rate (beats per minute)	61.43 ± 7.909	64.90 ± 8.608	< 0.001
HOMA-IR	1.016 ± 0.421	2.901 ± 1.521	< 0.001
Glucose (mg/dL)	93.04 ± 12.98	107.7 ± 21.79	< 0.001
Insulin (µIU/mL)	4.407 ± 1.753	10.98 ± 5.235	< 0.001
HbA1c (%)	5.524 ± 0.441	5.814 ± 0.578	< 0.001
Albumin (g/dL)	4.475 ± 0.229	4.517 ± 0.241	0.041
AST (IU/L)	20.40 ± 7.972	26.20 ± 11.02	< 0.001
ALT (IU/L)	18.83 ± 12.62	34.73 ± 24.79	< 0.001
Triglycerides (mg/dL)	104.5 ± 58.55	170.3 ± 103.3	< 0.001
Total cholesterol (mg/dL)	193.1 ± 33.50	197.7 ± 37.83	0.152
HDL-C (mg/dL)	58.96 ± 14.78	48.16 ± 12.96	< 0.001
LDL-C (mg/dL)	115.7 ± 30.10	121.0 ± 33.19	0.049
Systolic BP (mmHg)	107.5 ± 12.55	116.0 ± 13.09	< 0.001
Diastolic BP (mmHg)	69.75 ± 9.636	75.75 ± 9.717	< 0.001

*ALT Alanine aminotransferase, *AST* Aspartate aminotransferase, *BMI* Body mass index, *BP* Blood pressure, *HbA1c* Hemoglobin A1c, *HDL-C* High-density lipoprotein cholesterol, *HOMA-IR* Homeostatic model assessment of insulin resistance index, *LDL-C* Low-density lipoprotein cholesterol.

### Subject demographics

Among the 777 participants in the study, IS and IR included 611 and 166 individuals, respectively. Men accounted for 55.97% of IS and 84.94% of the IR group. The IS group had significantly lower values than those of the IR group for age, body mass index (BMI), waist circumference, heart rate, HOMA-IR, glucose, insulin, HbA1c, albumin, aspartate aminotransferase, alanine transaminase, triglycerides (TG), low-density lipoprotein cholesterol (LDL-C), and both diastolic and systolic blood pressure (BP). The IS group had lower total cholesterol than the IR group, but the difference was not statistically significant. Moreover, participants of IS group had higher high-density lipoprotein cholesterol (HDL-C) levels than those of the IR group with statistical significance (Table 1).

### Microbiome comparison and classification between the groups with or without FL

**Figure 2:**
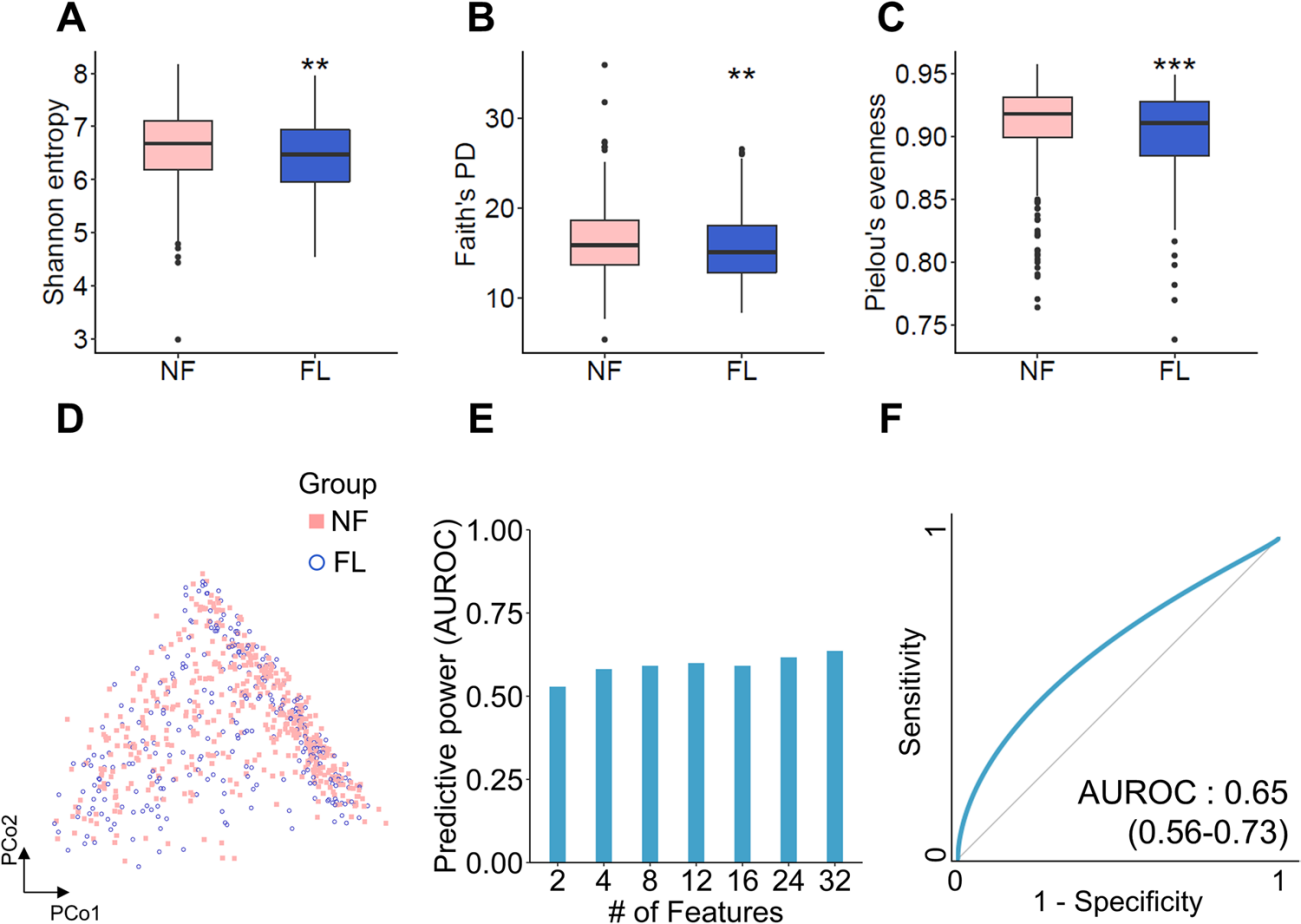
Comparison of the alpha and beta diversity of gut microbiome between fatty liver disease (FL) and nonfatty liver control (NF) groups. (**A**–**C**) Alpha diversity of NF and FL was measured using Shannon’s entropy (**A**), Faith’s phylogenetic diversity (PD) (**B**), and Pielou’s evenness (**C**). Boxes represent the IQR, whereas the upper whiskers represent the range from minimum (upper quartile − 1.5IQR) to maximum (lower quartile + 1.5IQR), and black dots represent outliers excluded in the range. (D) Beta diversity among participants in NF and FL was measured using the Principal Coordinates Analysis (PCoA). (E–F) The predictive power (AUROC) of the RF prediction model featuring different discriminative gut microbial genera in the training dataset (E) and test dataset (F). Statistical significance was analyzed using the Kruskal–Wallis test. *p < 0.05, **p < 0.01. FL: participants with fatty liver disease; IQR: interquartile range; ML: machine learning; NF: nonfatty liver control group; RF: random forest.

Alpha diversity, particularly Shannon’s entropy, was used to compare the diversity of the gut microbiome in participants of the non-fatty liver control group (NF) and FL groups. For Shannon’s entropy, representing biodiversity integrated with community richness and evenness, NF (median = 6.677; interquartile range [IQR] = 6.179–7.103) had a significantly higher value than that of FL (median = 6.475; IQR = 5.961–6.941; *p* = 0.0017; Fig. 2a). Consistent with previous reports, FL (median = 15.090, IQR = 12.859–18.057) had a significantly lower value of Faith’s phylogenetic diversity (PD: biodiversity based on phylogeny) than that of NF (median = 15.848, IQR = 13.648–18.594) (*p* = 0.004; Fig. 2b). Additionally, FL (median = 0.910; IQR = 0.885–0.928) had a lower value of Pielou’s evenness (a measure of biodiversity and species richness) than that of NF (median = 0.918; IQR = 0.899–0.931; *p* = 0.00045; Fig. 2c). Subsequently, we performed principal coordinate analysis (PCoA) to obtain representative relationships between the NF and FL groups. However, the two groups had no observable distant clusters (Fig. 2d).

As NF and FL showed differential alpha diversity but not beta diversity, we generated a classification model with informative gut microbial features. We can perform Gini importance-based core informative feature selection based on the algorithm. The predictive power of the models from the training dataset with two, four, eight, 12, 16, 24, and 32 features were 0.53, 0.59, 0.60, 0.59, 0.62, and 0.64 of AUROC, respectively (Fig. 2e). The FL prediction using the model featuring 32 gut microbial features displayed 0.65 (0.56–0.73) of AUROC in the test dataset (Fig. 2f). This implies an inefficient classification based on a set of most informative features between the NF and FL groups.

### Classification between NF and FL using reported gut microbial features

Recent reports have proposed a novel microbiome-based diagnostic tool for liver cirrhosis, the most advanced FL^13^. We built an RF classifier using gut microbial markers to test whether the markers could distinguish between FL and NF in our data. Differential abundance of gut microbial genera features, including *Acidaminococcus* spp., *Alistipes* spp*.*, *Bacteroides* spp., *Dorea* spp., *Enterobacter* spp*., Escherichia-Shigella* spp., *Eubacterium* spp., *Faecalibacterium* spp., *Klebsiella* spp., *Ruminococcus* (gnavus group) spp., *Streptococcus* spp., and *Veillonella* spp., were selectively observed between FL and NF (Supplementary Fig. 1a). Among these features, *Acidaminococcus* spp. (*p* = 8.9e−05)*, Alistipes* spp. (*p* = 7.5e−07)*, Faecalibacterium* spp. (*p* = 0.011)*,* and *Ruminococcus* spp. (gnavus group; *p* = 0.0018) had significantly different abundances in NF and FL, consistent with previous studies*.* Furthermore, we evaluated the sensitivity and selectivity of a set of these features using the AUROC. However, the predictive power of each model with different number of features was insufficient to distinguish between NF and FL (0.60 AUROC for a 12-feature model, 0.60 AUROC for an 8-feature model, 0.56 AUROC for a 6-feature model, and 0.56 AUROC for 4-feature model; Supplementary Fig. 1^1^).

### Microbiome comparison and classification between IRNF and IRFL

**Figure 3:**
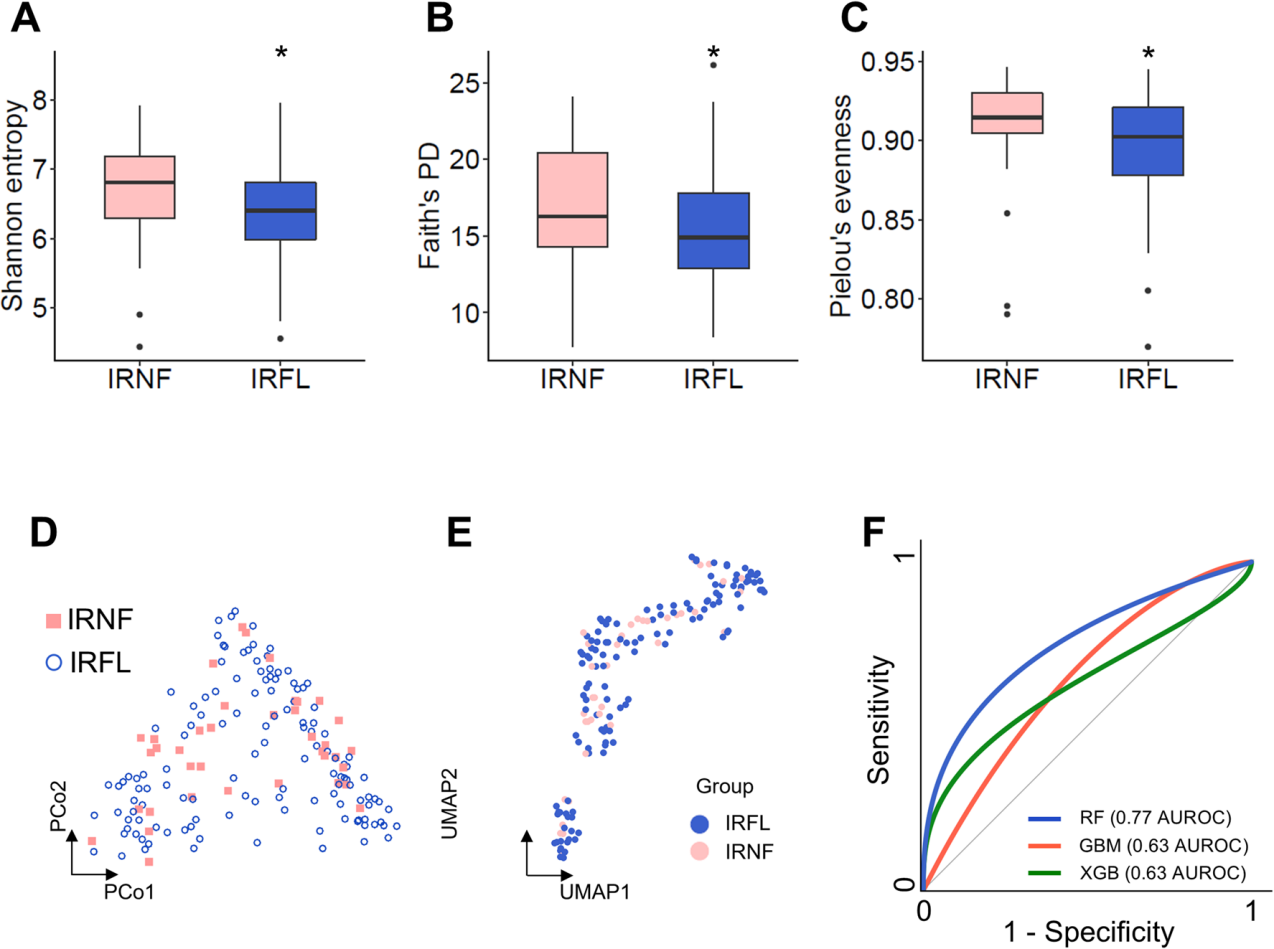
Comparing the alpha and beta diversity of the gut microbiome of IRNF and IRFL. (**A**–**C**) Alpha diversities of IRNF and IRFL were measured using Shannon’s entropy (**A**), Faith’s phylogenetic diversity (PD) (**B**), and Pielou’s evenness (**C**). Boxes represent the IQR. The upper whiskers represent the range from minimum (upper quartile − 1.5IQR) to maximum (lower quartile + 1.5IQR), and black dots represent outliers excluded in the range. (D–E) Beta diversity among participants in IRNF and IRFL was measured using PCoA (D) and UMAP analyses (E). (F) The model’s predictive power featuring different number of gut microbial genera constructed using RF, GBM, and XGB algorithms in the test dataset. The statistical significances were analyzed using Wilcoxon’s test. *p < 0.05, **p < 0.01. IRFL, fatty liver participants featuring insulin resistance; IQR: interquartile range; IRNF: nonfatty liver control group featuring insulin resistance.

NAFLD is considered the hepatic component of IR. Therefore, it is critical to distinguish FL from NF in participants with IR. The participants in the IR groups were divided into the following based on the presence of FL: NF featuring IR (IRNF) and FL featuring IR (IRFL), to find the most informative microbial features differentiating FL from NF in the participants with IR. Then, we observed the differential biodiversity of the two microbiomes. IRNF (median = 6.808, IQR = 6.289–7.183) had a significantly higher value of the index than that of IRFL (median = 6.403, IQR = 5.988–6.805; *p* = 0.032) in terms of Shannon’s entropy (Fig. 3a). Additionally, IRFL (median = 14.906, 12.876–17.804) had significantly lower Faith’s PD than that of IRNF (median = 16.293, IQR = 14.311–20.455; *p* = 0.032; Fig. 3b). In terms of other alpha diversity indices, IRFL (median = 0.902, IQR = 0.878–0.921) had lower Pielou’s evenness than that of IRNF (median = 0.915, IQR = 0.905–0.930; *p* = 0.021; Fig. 3c). However, PCoA and uniform manifold approximation and projection (UMAP) of the total microbiome of both groups showed no difference in clusters between IRFL and IRNF (Fig. 3d, e, Supplementary Fig. 2a and b).

To classify IRFL and IRNF from their gut microbiome, we constructed ML models using three ML algorithms for classification, RF, GBM, and XGB. Among the three ML models featuring different numbers of gut microbial genera, the RF model demonstrated the most reliable prediction in the test dataset (AUROC 0.77), while the AUROCs of classification for the other two ML models, GBM and XGB, were 0.62 and 0.63, respectively (Fig. 3f). Next, we built the models in the same manner, but individually for each gender, to see if any gender had better predictive results. In the training dataset, the RF model for females displayed AUROC values of 0.81, 0.96, 0.88, and 0.73 for the models using six-, eight-, twelve-feature, and entire gut microbiome, respectively (Supplementary Fig. 2c). The predictive power of the eight-feature model showed 0.67 AUROC. Surprisingly, the aforementioned outcome in the training dataset was superior to the results from the RF model for male, presenting AUROC values of 0.63 (six-feature model), 0.76 (eight-feature model), 0.69 (twelve-feature model), and 0.58 (entire gut microbiome-based model) (Supplementary Fig. 2d). During model validation using the male test dataset, the RF model had an AUROC of 0.76, the GBM model had an AUROC of 0.62, and the XGB model had an AUROC of 0.77 (Supplementary Fig. 2e). Together, it was determined that the models using the RF algorithm are appropriate for further research.

Then, we built RF models and assessed their efficacy in predicting FL in the IR groups after applying the SMOTE algorithm to the dataset to minimize the present class imbalance (30% IRNF: 70% IRFL). The model’s predictive power was 0.87 AUROC in the training dataset, but it only displayed 0.72 AUROC in the test dataset (Supplementary Table S1).

### Classification between IRNF and IRFL by using GA-optimized classifier (IRFL-GARF classifier)

GA is a heuristic algorithm that determines the global optimum based on natural selection^30–32^. It can be used to select model features such that the model demonstrates the best prediction. We used GA to create an ML classifier with better prediction performance using the RF algorithm. We developed an RF classifier presenting higher accuracy in distinguishing IRFL from IRNF, based on the features selected by GA. The RF classifier optimized by GA was termed “IRFL-GARF classifier,” with the potential gut microbial biomarkers^33^. Using the fitness score, the classifier can repeatedly search for the best solution for classifying IRFL and IRNF every generation.

**Figure 4:**
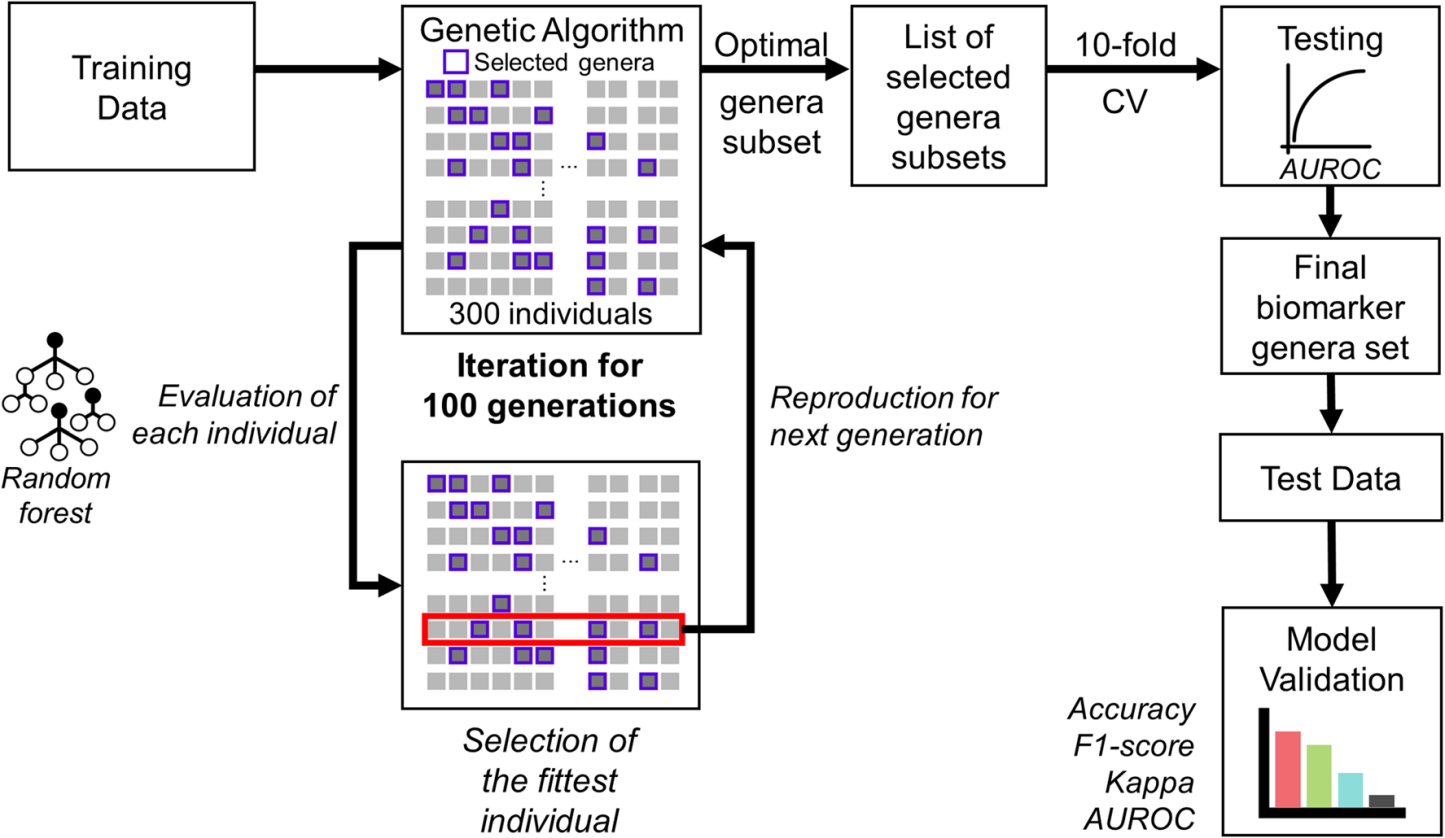
The overview of biomarker genera mining using GA. From the randomly generated initial 300 individuals consisting of genera, the classification model was optimized using GA methods, including crossover and mutation. The model with the highest fitness score is selected for each generation and further sequentially optimized in the next generation. The final model was evaluated using the average AUROC of the tenfold CV model. Further model validation was conducted using test data for the corresponding biomarker subset and accuracy, an F1-score, a kappa, and an AUROC.

In the development of the IRFL-GARF classifier, we first generated 300 individuals to be evolved further as the initial population (Fig. 4). Then, the fittest individual was selected following evaluation based on the fitness scores of every individual. With the fittest individual, the next generation is produced with crossover and mutation (Supplementary Fig. 3).

**Figure 5:**
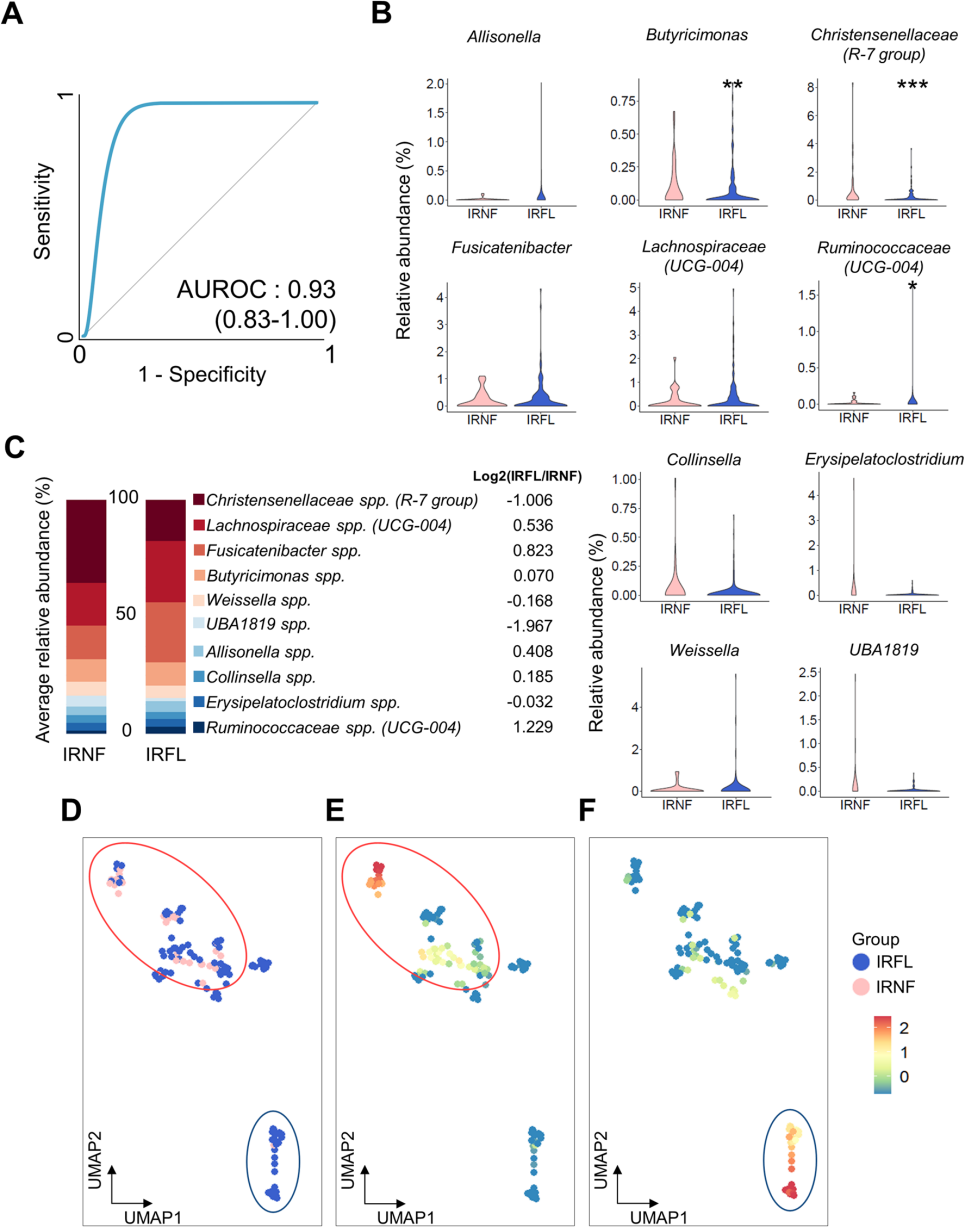
Prediction of FL in the presence of insulin resistance using GA-optimized classifier. (**A**) The predictive power (AUROC) derived from the test dataset using GA-optimized RF classifier with ten features. (**B**) Violin plots displaying relative abundances of core informative features in IRNF and IRFL. (**C**) Average relative abundances of discriminative features in the 10-feature prediction model in IRNF and IRFL. (**D**–**F**) UMAP analysis and heatmap of *Christensenellaceae* (R-7 group) spp. (**E**) and *Lachnospiraceae* (UCG-004) spp. onto UMAP. *p < 0.05, **p < 0.01, and ***p < 0.001 (Wilcoxon’s test). IRFL: fatty liver participants featuring insulin resistance; IRNF: nonfatty liver control group featuring insulin resistance; UMAP: uniform manifold approximation and projection.

Consequently, the GA reported ten optimal features (equivalently, genera) for an optimal RF model: *Christensenellaceae* (R-7 group) spp.*, Lachnospiraceae* (UCG-004) spp.*, Fusicatenibacter* spp., *Butyricimonas* spp.*, Weissella* spp.*, Ruminococcaceae* (UCG-004) spp.*, Erysipelatoclostridium* spp.*, UBA1819* spp.*, Allisonella* spp.*,* and *Collinsella* spp. The classifier model’s predictive power was 0.93 in the test dataset (95% confidence interval: 0.83–1.00; Fig. 5a). Between gut microbial features in the classifier model, *Butyricimonas* spp*.* (mean of IRNF: 0.111%; IRFL: 0.070%)*, Christensenellaceae (R-7 group)* spp. (IRNF: 0.736%; IRFL: 0.202%), *Collinsella* spp. (IRNF: 0.052%; IRFL: 0.021%), *Erysipelatoclostridium* spp. (IRNF: 0.153%; IRFL: 0.020%), and *UBA1819* spp. (IRNF: 0.104%; IRFL: 0.014%) displayed higher relative abundances in IRNF than in IRFL. In contrast, *Allisonella* spp. (IRNF: 0.008%; IRFL: 0.049%), *Fusicatenibacter* spp. (IRNF: 0.216%; IRFL: 0.305%), *Lachnospiraceae (UCG-004)* spp. (IRNF: 0.219%; IRFL: 0.358%), *Ruminococcaceae (UCG-004)* spp. (IRNF: 0.020%; IRFL: 0.026%)*,* and *Weissella* spp. (IRNF: 0.089%; IRFL: 0.117%) were more abundant in IRFL than in IRNF. Notably, *Butyricimonas* spp. (*p* = 0.0094), *Christensenellaceae (R-7 group)* spp. (*p* = 0.00056), and *Ruminococcaceae (UCG-004)* spp. (*p* = 0.026) had significantly different relative abundances between the two groups (Fig. 5b). The visualization of fold change in ten GA-selected features in the rate per hundred showed that *Christensenellaceae (R-7 group)* spp. (fold change of log2 [log2FC]: − 1.006), *Weissella* spp. (log2FC: − 0.168), *UBA1819* spp. (log2FC: − 1.967), *Collinsella* spp. (log2FC: − 0.185), and *Erysipelatoclostridium* spp. (log2FC: − 0.032) had lower relative abundances in IRFL. In contrast, *Lachnospiraceae (UCG-004)* spp. (log2FC: 0.536), *Fusicatenibacter* spp. (log2FC: 0.823), *Butyricimonas* spp. (log2FC: 0.070), *Allisonella* spp. (log2FC: 0.408), and *Ruminococcaceae (UCG-004)* spp. (log2FC: 1.229) were more abundant in both groups (Fig. 5c). Then, we performed UMAP projection to dimensionally reduce the dataset, presenting an IRNF clustering. *Christensenellaceae* spp*.* (R-7 group) were highly distributed in the green circle, where most IRNFs were distributed, whereas the *Lachnospiraceae* (UCG-004) group was highly distributed in the purple circle, where most of the dots represent IRFL (Fig. 5d–f).

Also, we developed a GA-optimized classifier for IS (a classification between IS participants without FL, ISNF, and IS participants with FL, ISFL). The model featured eight gut microbial genera, namely, *Eubacterium* spp. (coprostanoligenes group)*, Alistipes* spp., *Bifidobacterium* spp*., Erysipelotrichaceae* spp*.* (UCG-003), *Lachnoclostridium* spp.*, Parabacteroides* spp*., Ruminococcus* spp. (torques group), and *Subdoligranulum* spp*.* However, the model’s predictive power was insufficient to classify ISNF and ISFL (0.52 of an AUROC; Supplementary Fig. 4a and b).

### Model evaluation

**Figure 6:**
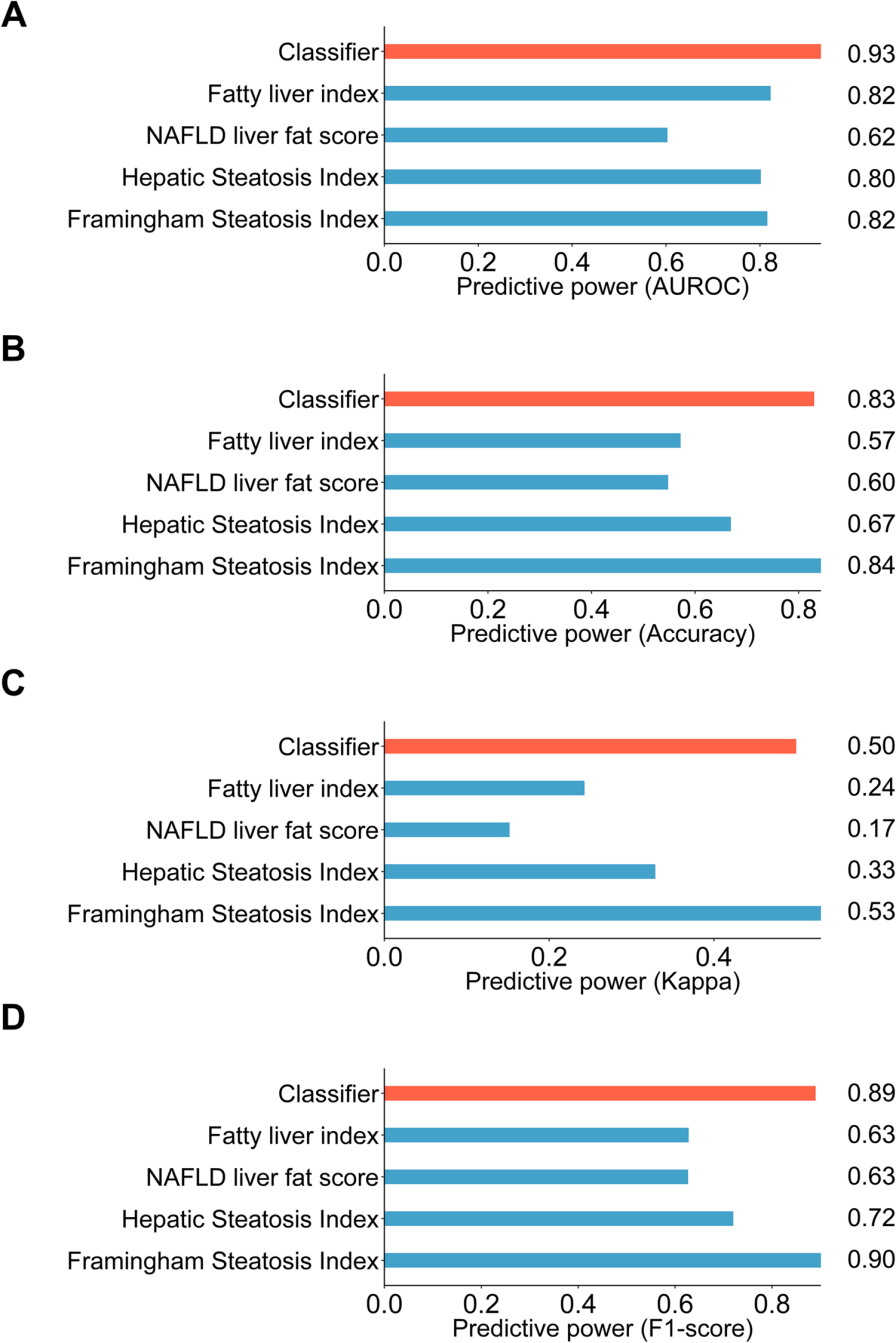
Evaluating prediction using GA-optimized classifier differentiating IRFL from IRNF. Bar plots comparing the predictive power derived from the test dataset using the GA-optimized classifier with other predictors by (**A**) AUROC, (**B**) accuracy, (**C**) kappa, and (**D**) F1 score. IRFL: fatty liver participants featuring insulin resistance; IRNF: nonfatty liver control group featuring insulin resistance.

To assess the GA-optimized model’s performance in IR, the model’s predictive power was compared with previously and broadly used non-invasive indexing scores calculated from clinical data for predicting FL, including FL index (FLI)^34^, NAFLD liver fat score (NAFLD-LFS)^35^, hepatic steatosis index (HSI)^36^, and Framingham steatosis index (FSI)^37^. For comparison, each score was calculated for each IR analyzed for the study and used for FL prediction with a partitioned test dataset. Our classifier displayed 0.93 AUROC, as the FLI, NAFLD-LFS, HSI, and FSI values were 0.82, 0.62, 0.80, and 0.82, respectively (Fig. 6a). The prediction accuracies of the GA-optimized classifier, FLI, NAFLD-LFS, HSI, and FSI were 0.83, 0.57, 0.60, 0.67, and 0.84, respectively (Fig. 6b). Additionally, the FL prediction by our classifier presented a kappa of 0.50, while the kappa of FLI, NAFLD-LFS, HSI, and FSI were 0.24, 0.17, 0.33, and 0.53, respectively (Fig. 6c). Finally, our classifier displayed 0.89 F1-score, which was similar to the FSI (0.90), whereas FLI, NAFLD-LFS, and HSI displayed 0.63, 0.63, and 0.72 of F1-scores, respectively (Fig. 6d). As shown above, among all measuring methods for predicting the power of predictors, our classifier gave the highest diagnostic accuracy compared with other predictors. This result implied that our gut microbiome-based classifier could be used with the abovementioned established predictors.

## Discussion

In this study, we performed an RF classification model using the KSH cohort comprising 777 healthy individuals, and we applied GA to improve the predictive efficiency of the RF-generated classifier (an AUROC of 0.77) (Fig. 3f). Based on the IRFL-GARF, we proposed ten genera as biomarkers for identifying FL in IR individuals, including *Christensenellaceae* (R-7 group) spp., *Weissella* spp., *UBA1819* spp., *Collinsella* spp., *Erysipelatoclostridium* spp.*, Lachnospiraceae* (UCG-004) spp., *Fusicatenibacter* spp., *Butyricimonas* spp., *Allisonella* spp., and *Ruminococcaceae* (UCG-004) spp. The IRFL-GARF classifier containing ten microbial genera had an AUROC, accuracy, kappa, and F1-score of 0.93, 0.83, 0.50, and 0.89, respectively, which are comparable or even superior to common diagnostic indices for FL diseases, such as FLI (AUROC of 0.82), HSI (AUROC of 0.80), NAFLD-LFS (AUROC of 0.60), and FSI (AUROC of 0.82). This demonstrates the potential of a diagnostic marker for FL disease in insulin-resistant participants (Fig. 6a–d).

GA is an adaptive metaheuristic search algorithm that identifies the global optimum based on the principle of natural selection in evolution. GA does not evaluate solutions individually but evaluates a group of solutions simultaneously and explores the space of possible solutions. Furthermore, GA has the advantage of being less likely to fall into a local minimum and does not require assumptions about the interaction between features.

The ten genera could be developed into a non-invasive biomarker for FL disease in insulin-resistant participants, who could have a higher chance of developing advanced chronic liver diseases. To the best of our knowledge, this is the first study that used GA to successfully classify FL diseases, encouraging GA application in future studies. The development of a non-invasive, inexpensive, and accurate method for diagnosing FL disease is required. Several recent studies have proposed the gut microbiome as a potential biomarker for advanced chronic liver diseases^13–15,38^; however, only a few studies have been conducted in generally healthy populations to identify individuals at higher risk of developing advanced chronic liver diseases based on the gut microbiome. For instance, it would be important to identify generally healthy participants showing IR without symptoms with a higher risk of advanced FL diseases.

In this study, alpha diversity, which reflects the gut microbiome structure concerning its richness^39^, decreased significantly in the FL participants. In contrast, the PCoA plots, representing beta diversity of the gut microbiome, failed to generate two distinct groups, inconsistent with previous studies^13,38^. Estimating alpha and beta diversities implies that reduced richness was sufficient to show differences in alpha diversity between the groups. However, it occurred in only a few genera (components). Although it is insufficient to determine whether the altered genera drove FL or vice versa, several studies have reported that the family *Christensenellaceae* correlates with BMI^40^, and the *Ruminococcaceae* (*R-7 group*) genera correlate with blood TG, very-low-density lipoprotein- and HDL-particles levels^41^. Another recent human study observed a strong correlation between *Collinsella* spp. and NASH and cirrhosis^42^. Few studies have indicated that *Butyricimonas* spp. is altered in AFLD and HCC^43,44^, implying that *Butyricimonas* spp. might contribute to FL disease or hepatic inflammation.

There are a few limitations to our study. Firstly, our research was based on a Korean hospital cohort with not quite large patients; thus, our results could be racially and geographically biased. However, we tried to prove the feasibility of the discovered ten genera as a biomarker to identify FL disease among patients with IR from the supporting studies. Secondly, it was implied that predicting FL using gut microbiome-based ML in the female IR groups could be more reliable rather than in the male group. However, greater sample size is needed for further validation. Additionally, to our knowledge, there was no independent external validation cohort available; thus, we expect that further validation studies population will address these limitations. Although our study is primarily correlative, our data strongly support the value of future work exploring the causal role of the ten genera in liver diseases.

Conclusively, these findings indicate that the ML classifier combined with GA for FL in the presence of IR is comparable or superior to commonly used methods. The ten genera we discovered are useful as a non-invasive biomarker for FL among patients with IR.

## Supplementary Information

Supplementary Information 1.

Supplementary Information 2.

Supplementary Information 3.

Supplementary Information 4.

Supplementary Information 5.

## Data Availability

All 16S rDNA gene sequencing files are available in the Clinical & Omics Data Archive of the Korea National Institute of Health (http://coda.nih.go.kr; accession number: R000635). The source code for the GA simulation is available on GitHub (https://github.com/labnams/FLIRGAMB).
