# Supplementary figures and images for: Machine learning-derived gut microbiome signature predicts fatty liver disease in the presence of insulin resistance
Source: Sci Rep. 2022 Dec 17;12:21842. doi: 10.1038/s41598-022-26102-4 (PMC9759583; doi:10.1038/s41598-022-26102-4)

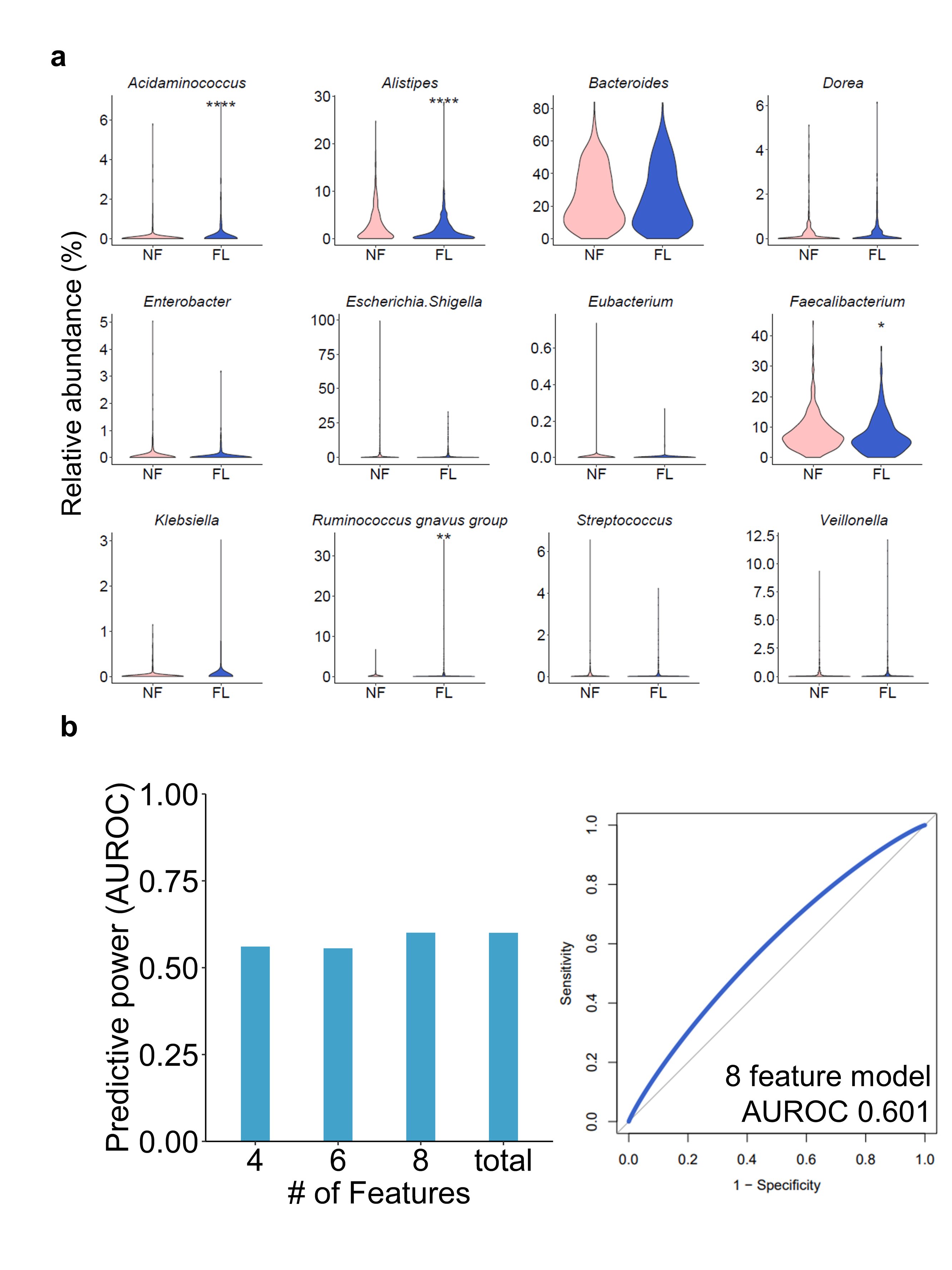

Supplement: Supplementary file 1 — Supplementary Information 1. [file 41598_2022_26102_MOESM1_ESM.tif]

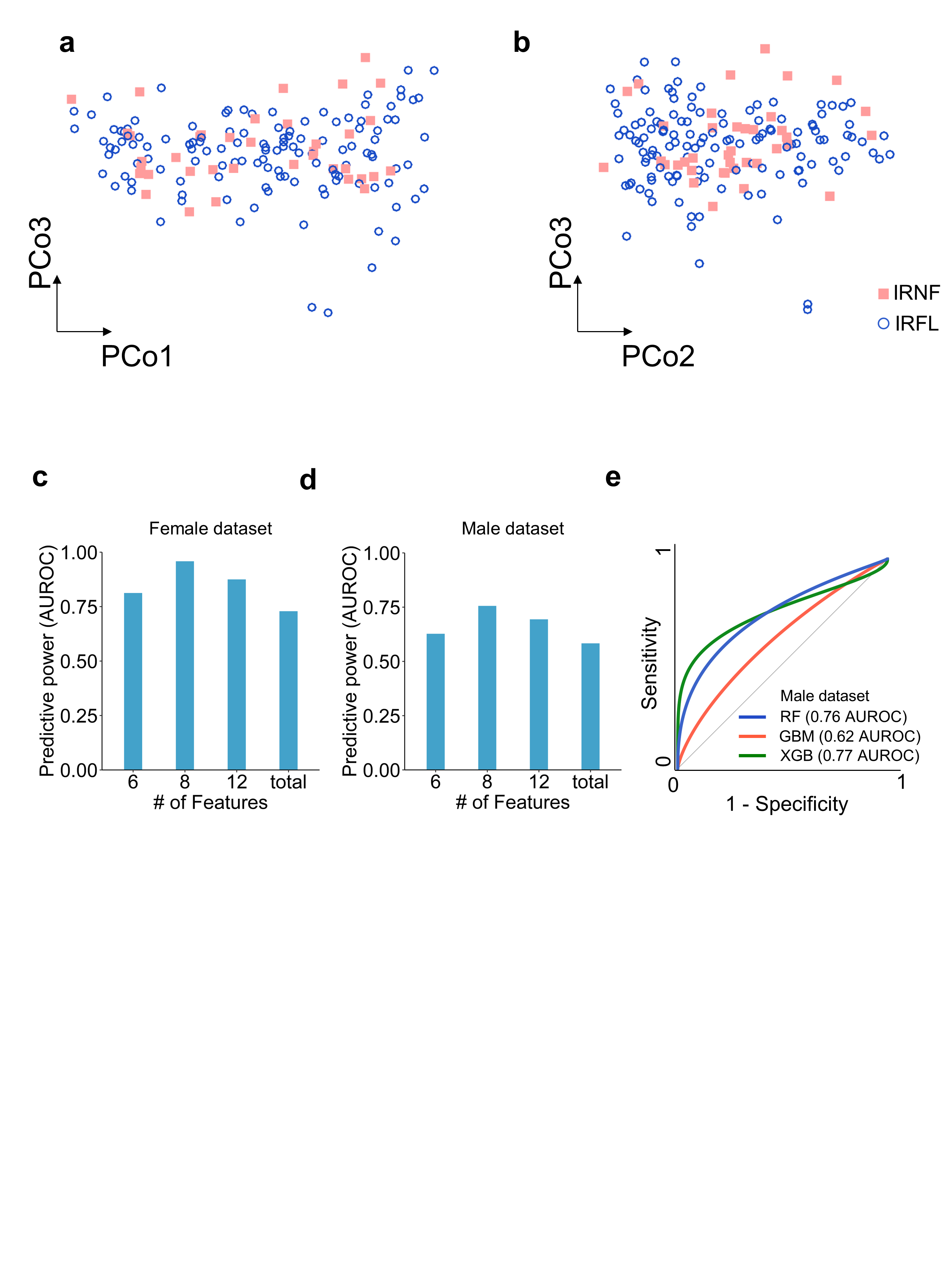

Supplement: Supplementary file 2 — Supplementary Information 2. [file 41598_2022_26102_MOESM2_ESM.tif]

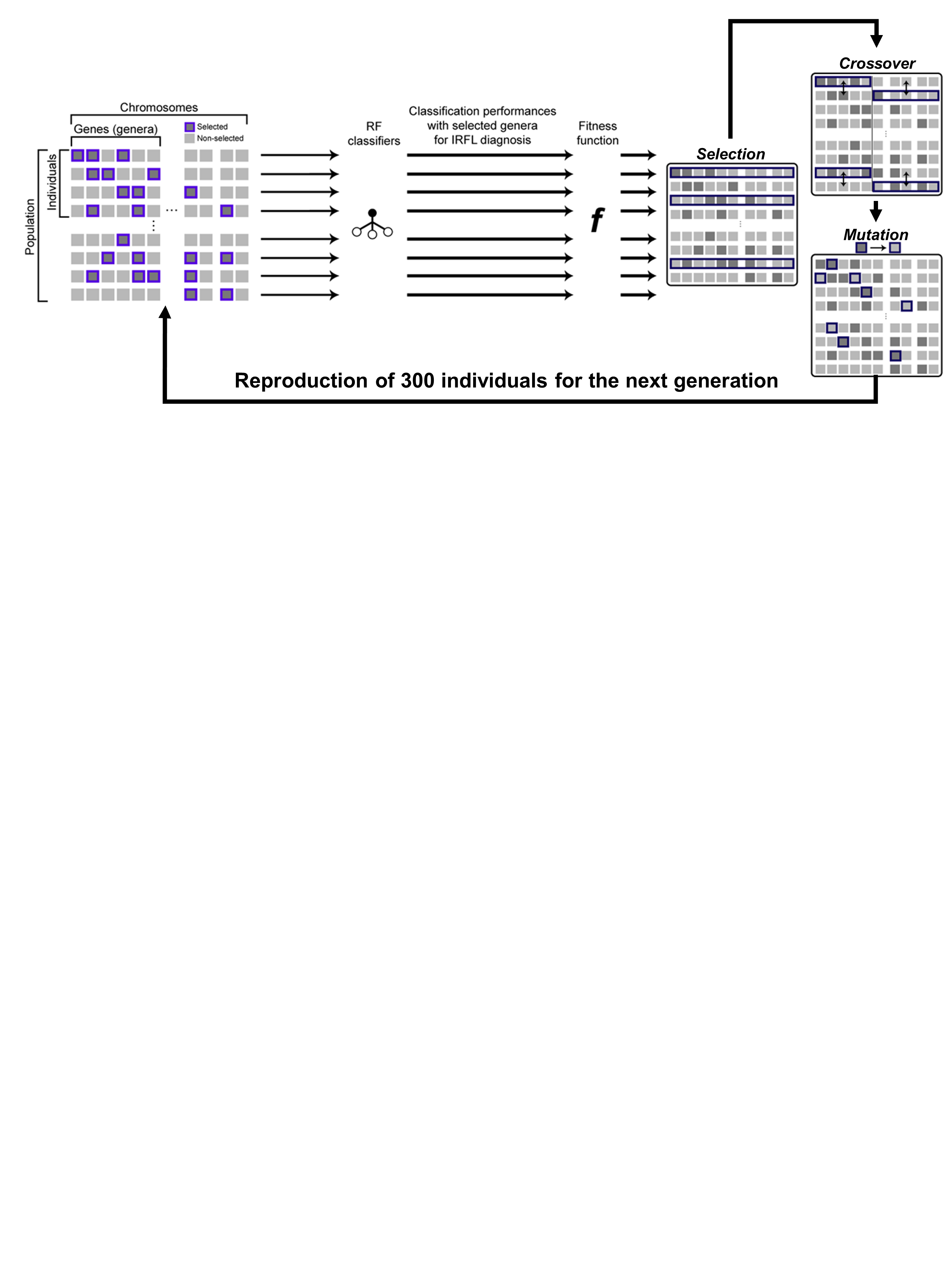

Supplement: Supplementary file 3 — Supplementary Information 3. [file 41598_2022_26102_MOESM3_ESM.tif]

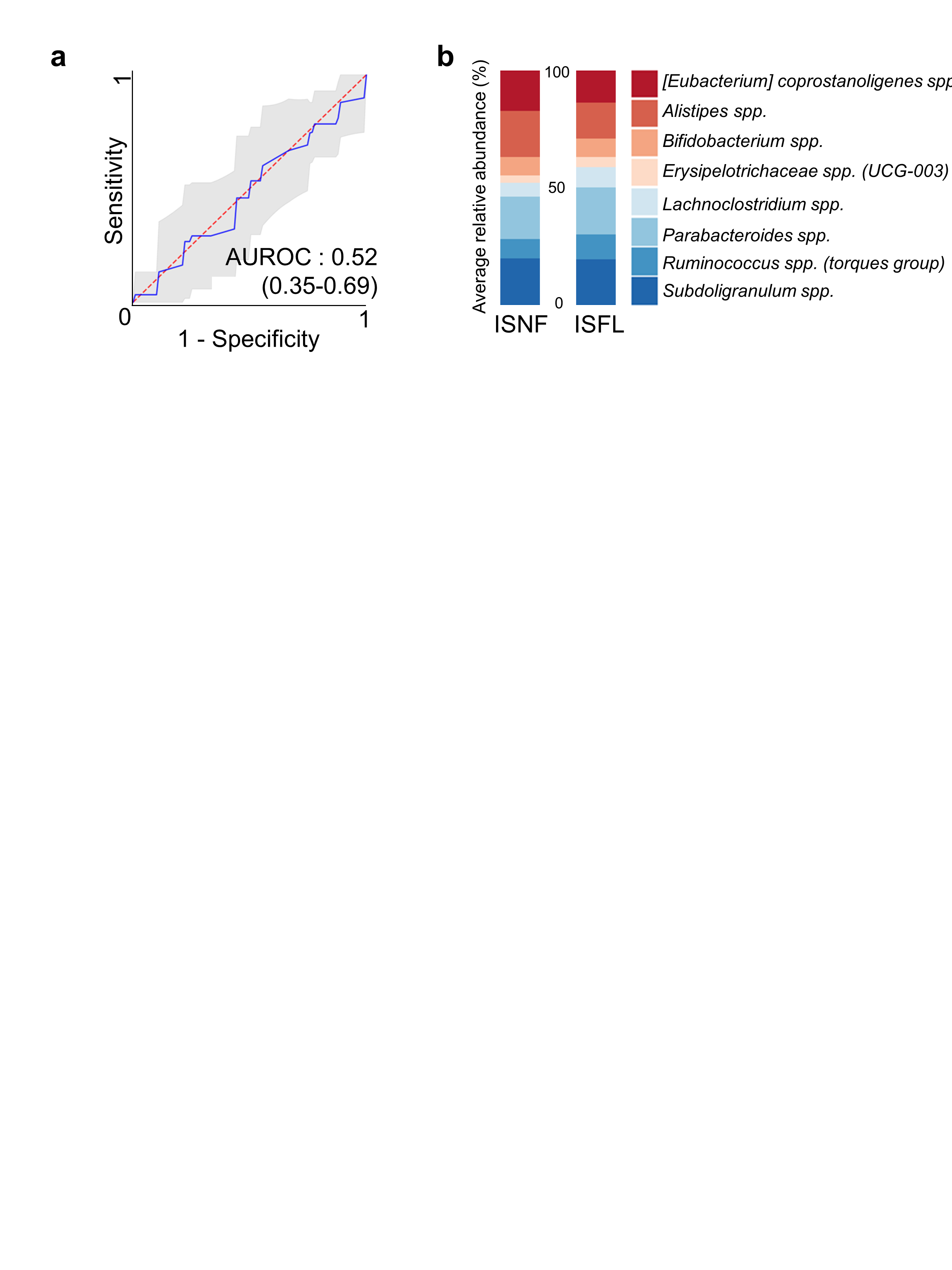

Supplement: Supplementary file 4 — Supplementary Information 4. [file 41598_2022_26102_MOESM4_ESM.tif]
